# Extended live-cell barcoding approach for multiplexed mass cytometry

**DOI:** 10.1038/s41598-021-91816-w

**Published:** 2021-06-11

**Authors:** Muharrem Muftuoglu, Li Li, Shaoheng Liang, Duncan Mak, Angelique J. Lin, Junxiang Fang, Jared K. Burks, Ken Chen, Michael Andreeff

**Affiliations:** 1 Section of Molecular Hematology and Therapy, Department of Leukemia, The University of Texas MD Anderson Cancer Center, Unit 448, 1515 Holcombe Blvd., Houston, TX 77030 USA; 2 Department of Lymphoma, University of Texas MD Anderson Cancer Center, Houston, USA; 3 Department of Bioinformatics and Computational Biology, University of Texas MD Anderson Cancer, Houston, USA; 4 Department of Computer Science, Rice University, Houston, USA; 5 Department of Statistics and Data Sciences, The University of Texas at Austin, Austin, USA

**Keywords:** Bioinformatics, Cytological techniques, High-throughput screening, Cancer, Molecular medicine, Oncology

## Abstract

Sample barcoding is essential in mass cytometry analysis, since it can eliminate potential procedural variations, enhance throughput, and allow simultaneous sample processing and acquisition. Sample pooling after prior surface staining termed live-cell barcoding is more desirable than intracellular barcoding, where samples are pooled after fixation and permeabilization, since it does not depend on fixation-sensitive antigenic epitopes. In live-cell barcoding, the general approach uses two tags per sample out of a pool of antibodies paired with five palladium (Pd) isotopes in order to preserve appreciable signal-to-noise ratios and achieve higher yields after sample deconvolution. The number of samples that can be pooled in an experiment using live-cell barcoding is limited, due to weak signal intensities associated with Pd isotopes and the relatively low number of available tags. Here, we describe a novel barcoding technique utilizing 10 different tags, seven cadmium (Cd) tags and three Pd tags, with superior signal intensities that do not impinge on lanthanide detection, which enables enhanced pooling of samples with multiple experimental conditions and markedly enhances sample throughput.

## Introduction

Flow cytometry has dominated single cell analysis for decades and has been an indispensable tool for the study of heterogeneous cell populations and complex biological systems. Mass cytometry, also known as cytometry by time-of-flight (CyTOF), has revolutionized the proteomics field and significantly expanded the scope of phenotypic and functional analyses at the single-cell level^1,2^. In contrast to conventional flow cytometry, CyTOF relies on the use of specific monomers or polymers functioning as metal chelators prior to antibody tagging. The number of analytes measured by CyTOF relies on isotope purification, design of novel chelators to capture different classes of metals, and the availability of appropriate chemical methods for antibody labeling. The mass window of current mass cytometers will theoretically allow assessment of 135 parameters per cells, which has triggered a growing interest to expand the range of parameters that can be assessed by CyTOF. The trial-and-error approach of assessing if mDTPA (maleimide-diethylenetriaminepentaacetic acid) polymers can capture non-lanthanide trivalent metals, such as two indium isotopes^3^, the modification of lanthanide antibody labeling protocols allowing antibody tagging of new metal isotopes^4^, the use of a new class of chelators to capture bivalent palladium isotopes^5–7^, the utilization of heavy-metal-containing compounds for antibody labeling^8^, and cell barcoding^9,10^ have all significantly expanded measurable parameters permitting assessment of > 50 per single cell^5^. The development of MCP9 (metal-chelating polymer 9) polymers to capture seven Cd isotopes further pushed the boundaries and thus enabled performing up to 60 measurements per single cell.

Sample multiplexing using various barcoding schemes to improve sample-to-sample consistency and overcome technical variations by permitting simultaneous processing and acquisition of pooled sample sets, in which a unique barcode is assigned to each sample, has been widely adopted. The current commercially-available barcoding kit also employs a Pd-based “intracellular” barcoding approach and enables multiplexing of up to 20 samples using a 6-choose-3 scheme^7^. However, intracellular barcoding requires sample fixation and permeabilization before sample barcoding and pooling, which is followed by surface and intracellular staining^11^. This method is not compatible and interferes with the detection of certain surface antigens, especially chemokine receptors^12^. On the other hand, live-cell barcoding allows sample pooling and surface staining before fixation, thereby preserving “fixation-sensitive” surface antigens. To date, Pd, indium (In) and platinum (Pt) tagged antibodies, which do not impinge on lanthanide detection, against ubiquitously expressed antigens such as CD45, beta-2-microglobuin (B2M), CD298, and HLA-ABC, and monoisotopic tellurium-based compounds^13^ have been explored for live-cell barcoding^5,14–16^. ITCBE (isothiocyanobenzyl-ethylenediaminetetraacetic acid)^16^ and mDOTA^5^ (maleimido-mono-amido-DOTA) monomers are capable of chelating bivalent Pd isotopes, and monomers loaded with Pd isotopes can be successfully tagged to CD45, though it is more labor-intensive compared to generation of lanthanide-tagged antibodies using readily available mDTPA polymers. Current live-cell barcoding approaches allow to barcode and pool up to 20 experimental conditions^13,14,16^. Conjugation using bivalent Pd isotopes loaded onto monomeric chelators necessitates lyophilization to remove solvents prior to antibody tagging, which significantly prolongs the conjugation process. Furthermore, Pd isotopes generate relatively weak signals as they are situated at the lower end of the sensitivity spectrum^7^. Weaker signals associated with Pd isotopes significantly limit the extent of barcoding schemes to be utilized, allowing use of only two tags per sample primarily.

The number of tags used in barcoding dictates the number of experimental designs that can be barcoded in various barcoding schemes. For example, using 6-choose-2 versus a 6-choose-3 barcoding scheme enables barcoding 15 and 20 samples, respectively. Consequently, it is particularly appealing to extend the barcoding scheme by incorporating more mass tags, thus ensuring barcoding a broader range of experimental designs. Inherently, Cd isotopes stand out as potential candidates that could be used for live-cell barcoding. Moreover, Cd isotopes are located well below the 139–176 mass range of lanthanides and do not influence lanthanide-based antibody detection through isotope impurity, abundance and oxidation. The recent introduction of MCP9 polymers could facilitate the utilization of Cd isotopes in CyTOF. Therefore, we designed a barcoding scheme comprising CD45 antibodies paired with seven Cd (i.e., 106Cd, 110Cd, 111Cd, 112Cd, 113Cd, 114Cd, and 116Cd) and three Pd (i.e., 104Pd, 105Pd, and 108Pd) isotopes. Both the Cd and Pd isotopes were loaded onto the MCP9 polymer after we demonstrated MCP9 polymer could capture bivalent Pd isotopes as well, and we used a 10-choose-2 combinatorial scheme. This live-cell barcoding platform considerably expanded our ability to pool and barcode higher numbers of samples, which we describe herein.

## Results

### Generation of Cd-tagged, CD45-based barcoding reagents

Sample barcoding and pooling prior to surface staining had been explored using CD45, B2M, and CD298 antibodies labeled with Pd and indium isotopes, and monoisotopic cisplatin compounds^5,14,16^. A major limitation of Pd-tagged antibodies is their generation of weak signals compared to antibodies labeled with lanthanide isotopes using mDTPA polymers, which significantly constrains the size of the mass-tag cell barcoding pool that can be used for live-cell barcoding. The newly developed MCP9 polymers (Fluidigm, San Francisco, CA), specifically if used to chelate seven bivalent Cd isotopes, could be potentially exploited to generate low-sensitivity metal-tagged antibodies with improved signal-to-noise ratio. We reasoned that MCP9-loaded Cd isotopes could be used to generate surface barcoding reagents through pairing with abundantly expressed antigens. To demonstrate proof of concept, we selected CD45 that is abundantly expressed on hematopoietic cells, although at varying abundance across different subsets, as a candidate to generate barcoding reagents.

**Figure 1:**
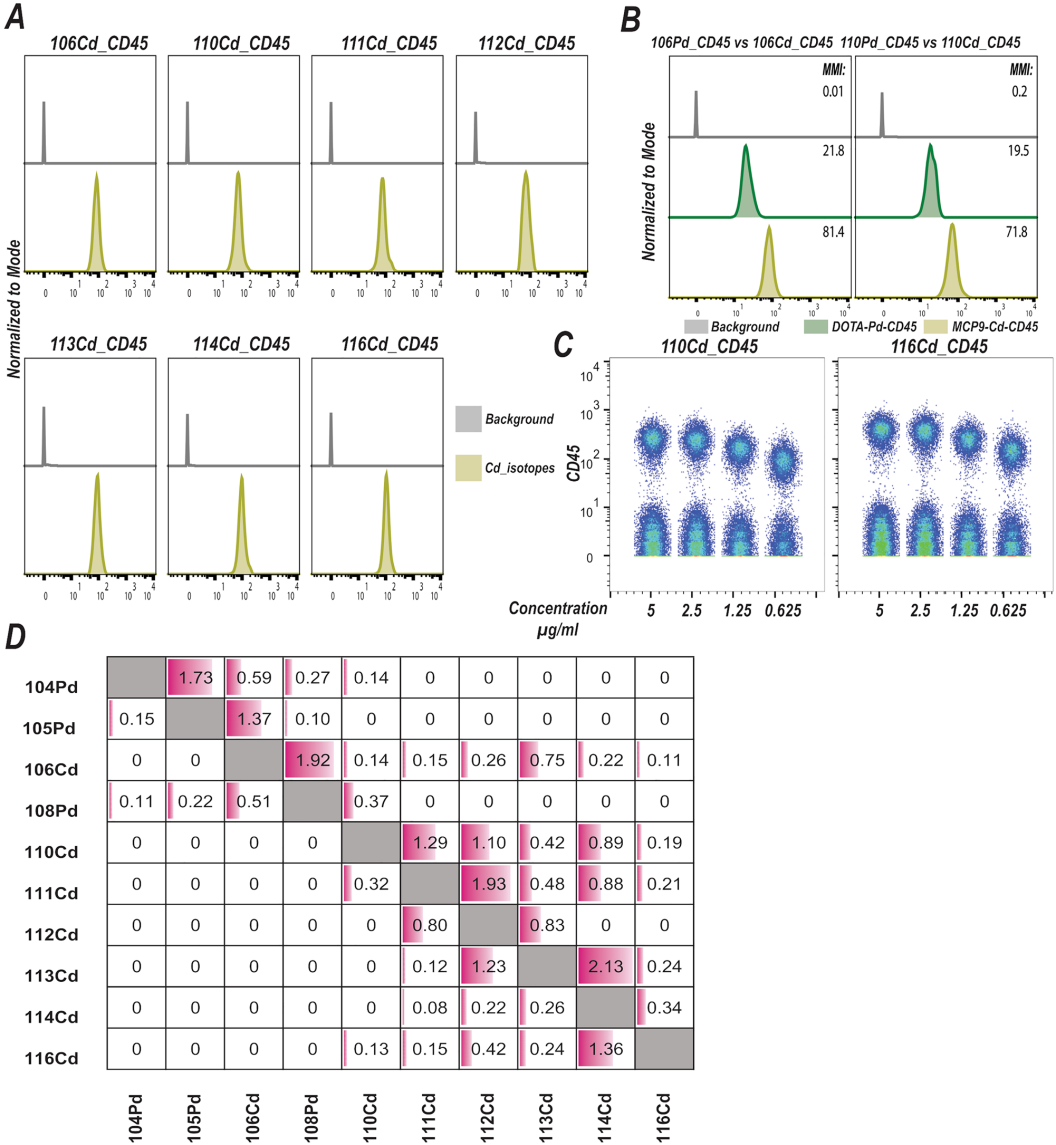
Cadmium-tagged CD45 antibodies elicit higher signal intensities. (**A**) Histograms show signal intensities for 7 Cd isotopes tagged to CD45 antibody (HI30) and antibody capture beads were used for signal quantification. Gray histograms show background signal intensities for each Cd isotopes. To estimate background signal intensities for a metal of interest we utilized capture beads stained with CD45 tagged to a Cd isotope other than the Cd isotope of interest. (**B**) Histograms show signal intensities for Pd and Cd isotopes with overlapping mass weights. Capture beads were labeled with equal amounts of CD45 antibodies tagged with MCP9-loaded Cd and mDOTA-loaded Pd isotopes. Gray histograms show background signal intensities of isotopes having mass weights of 106 and 110. (**C**) Biaxial plots show signal intensities for serial dilution of CD45 tagged to 110Cd and 116Cd isotopes using PBMCs. FCS files were concatenated after separate acquisition of each file and concatenated FCS files display serial dilution for two Cd isotopes tagged with CD45. (**D**) Spill-over matrix for 3 Pd and 7 Cd isotopes. The matrix is generated using antibody capture beads labeled with Cd and Pd isotopes separately. The numbers in the cells represent the percentage of spill-over.

We generated seven CD45-based barcoding reagents by pairing CD45 antibody (HI30) with seven MCP9-loaded Cd isotopes. The antibody yield after conjugation ranged from 60 to 70%. We also generated five CD45-mDOTA-Pd conjugates (i.e., 104Pd, 105Pd, 106Pd, 108pd, and 110Pd) for comparison and antibody recovery after tagging with Pd-loaded mDOTA monomers was approximately 80 to 90%. This was essentially due to the necessity to utilize different antibody purification methods as detailed in the method section. We reasoned that as a polymeric bifunctional chelator MCP9 could chelate and capture more bivalent ions compared to monomeric bifunctional chelator mDOTA, and thus generate higher signal intensities. Initially, we used antibody-labeled capture beads (see “Methods” section) to estimate the performance of the Cd-tagged CD45 antibodies in comparison to CD45 antibodies labeled with Pd isotopes loaded onto mDOTA. The capture beads labeled with CD45-tagged with 7 Cd isotopes generated appreciable signal intensities (Fig. 1A). Having two cadmium isotopes, 106Cd and 110Cd, overlapping with two Pd isotopes, 106Pd and 110Pd, enabled us to directly compare Cd isotopes loaded onto MCP9 polymer with corresponding Pd isotopes with similar mass loaded onto mDOTA monomer. Signal intensities associated with 106Cd and 110Cd-CD45 were significantly higher compared to 106Pd and 110Pd-CD45 antibodies, indicating that MCP9 polymers may be capable of chelating a higher number of bivalent metals than the mDOTA monomers and provide superior signal resolution (Fig. 1B).

Next, we labeled peripheral blood mononuclear cells (PBMCs) from healthy donors with individual Cd-CD45 antibodies to assess if Cd signal intensities measured by capture beads would translate to superior single intensities and signal-to-noise ratios when PBMCs were used for labeling. We first serially diluted seven Cd-tagged CD45 antibodies separately and individual samples labeled with identical concentrations of different Cd-tagged CD45 antibodies were pooled before acquisition. Staining with a fixed 2.5 µg/ml concentration produced acceptable signal-to-noise ratios for the majority of antibodies (Fig. 1C and Fig. S1A). Importantly, we observed background noise due to feedback from neighboring channels when samples were stained separately with individual barcoding tags and pooled before sample acquisition. This could potentially impinge on barcoding depth if a channel receives signals from other channels. Indeed, the 112Cd, 113Cd, and 114Cd channels received spill-over from multiple channels (Fig. 1D), resulting in increased background noise (Fig. 1C and Fig. S1A). This was mainly due to metal impurities (Fig. S1B). In this context, caution is warranted and the use of multiple tags could result in higher background noise, which could in turn limit the number of tags that could be used per sample. These findings indicate that MCP9 polymers can chelate higher numbers of metals relative to their monomeric counterparts, and provide markedly improved signal-to-noise ratios for metals situated at the lower end sensitivity spectrum.

### Pd labeling of antibodies using the MCP9 polymer

While mDTPA polymers have been specifically developed to chelate lanthanide metals, indium (In) isotopes, 113In and 115In, and the bismuth (Bi) isotope 209Bi can also be loaded onto mDTPA polymers and used for antibody tagging^4,17^. In a similar fashion, we reasoned that bivalent Pd isotopes could be loaded onto MCP9 polymers and these metal-polymer complexes could be then used for antibody tagging. As a proof-of-principle, we initially used natural abundance Pd(NO_3_)_2_ (palladium nitrate) dissolved in double distilled water (ddH_2_O) to 50 mM and tested if Pd metals could be loaded onto MCP9 polymers. To test whether MCP9 can chelate bivalent Pd ions we stained PBMCs with CD45 tagged to MCP9-loaded natural abundance Pd. Indeed, MCP9 polymer successfully captured Pd ions and signal intensities were in line with natural abundance of Pd isotopes (Fig. S2A).

**Figure 2:**
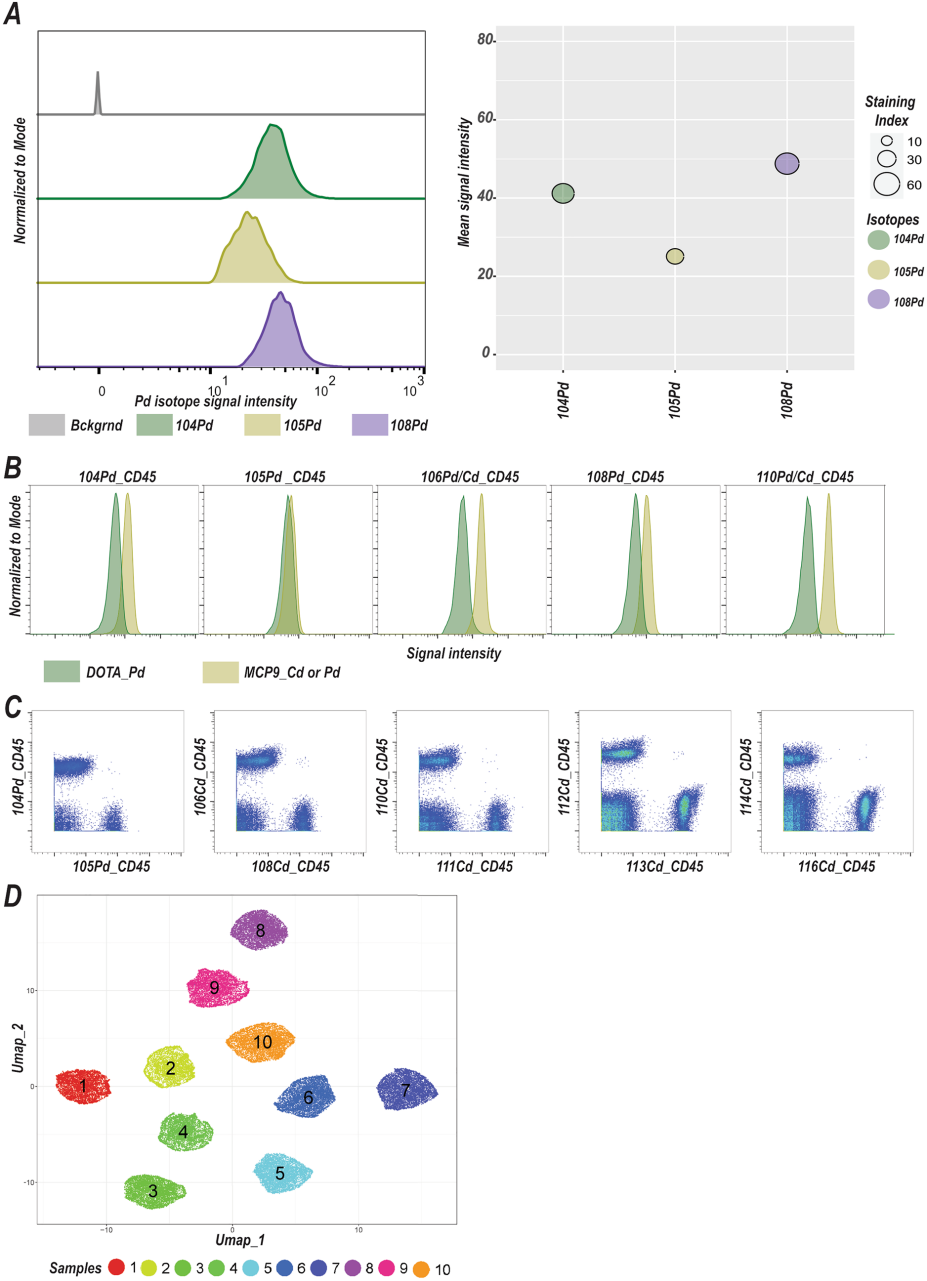
MCP9 polymer chelates Palladium isotopes. (**A**) Signal intensities for 3 MCP9-loaded Pd isotopes, 104Pd (green), 105Pd (yellow) and 108Pd (purple), tagged with CD45 (left panel). Gray histograms show background signal intensities. Capture beads are labeled with equal amounts of MCP9-loaded Pd isotopes tagged with CD45. The bubble plot shows mean signal intensities and staining indices for 104Pd (green), 105Pd (yellow), and 108Pd (purple) in A (right panel). (**B**) Histogram shows signal intensities for Cd and Pd isotopes loaded to MCP9 or mDOTA. Five Pd isotopes, 104Pd, 105Pd, 106Pd, 108Pd and 110Pd were loaded to mDOTA. For comparison 3 Pd, 104Pd, 105Pd, and 108Pd, and 2 Cd isotopes, 106Cd and 110Cd, were loaded to MCP9 polymer for CD45 antibody conjugation. PBMCs from healthy donors were stained with 10 different MCBs (mass-tag cell barcodes), 5 mDOTA-based and 5 MCP9-based, at a concentration of 2.5 µg/ml for each antibody. (**C**) Signal intensities for 10 MCBs are shown in biaxial plots. PBMCs from a healthy donor were separately labeled with 10 different MCBs utilizing MCP9 polymers loaded with 7 Cd and 3 Pd isotopes. Samples were then pooled and acquired simultaneously. (**D**) Pooled samples (n = 10) were subjected to dimension reduction and clustering using UMAP and FlowSOM algorithms, respectively. Each color code corresponds to a single sample.

Next, we loaded three Pd isotopes, 104Pd, 105Pd, and 108Pd onto MCP9, tagged Pd-MCP9 complexes to CD45 and then utilized antibody capture beads to assess metal loading and antibody tagging efficiency. Similarly, MCP9 polymers were able to successfully capture Pd metals (Fig. 2A). This led us to further extend our barcoding toolbox, which already included seven Cd-based barcoding reagents. Pd isotopes, 104Pd and 108Pd, loaded to MCP9 polymers generated higher signal intensities compared to their counterparts paired with mDOTA, while 105Pd paired with either MCP9 versus mDOTA resulted in similar signal intensities (Fig. 2B). Having established and validated that MCP9 can be used to chelate Pd isotope subsequently allowed us to use 10 different barcoding reagents for multiplexing and greatly extended our multiplexing capabilities.

We next labelled samples individually with different Cd or Pd tags and pooled 10 samples before acquisition to reduce technical variation, assess channel noise, and simulate a barcoding scheme that enabled assessment of barcode separation. Remarkably, we observed at least a log^10^ difference between CD45 + and CD45- populations for each individual, Cd-tagged antibody. The signal-to-noise ratio was less favorable for the Pd-tagged CD45 antibodies (Fig. 2C, Fig. S2B). Nevertheless, there was obvious segregation among pooled samples labeled with single tags in the high-dimensional space (Fig. 2D, Fig. S2C).

### Enhancement live-cell barcoding capability through the combined use of Pd and Cd isotopes

**Figure 3:**
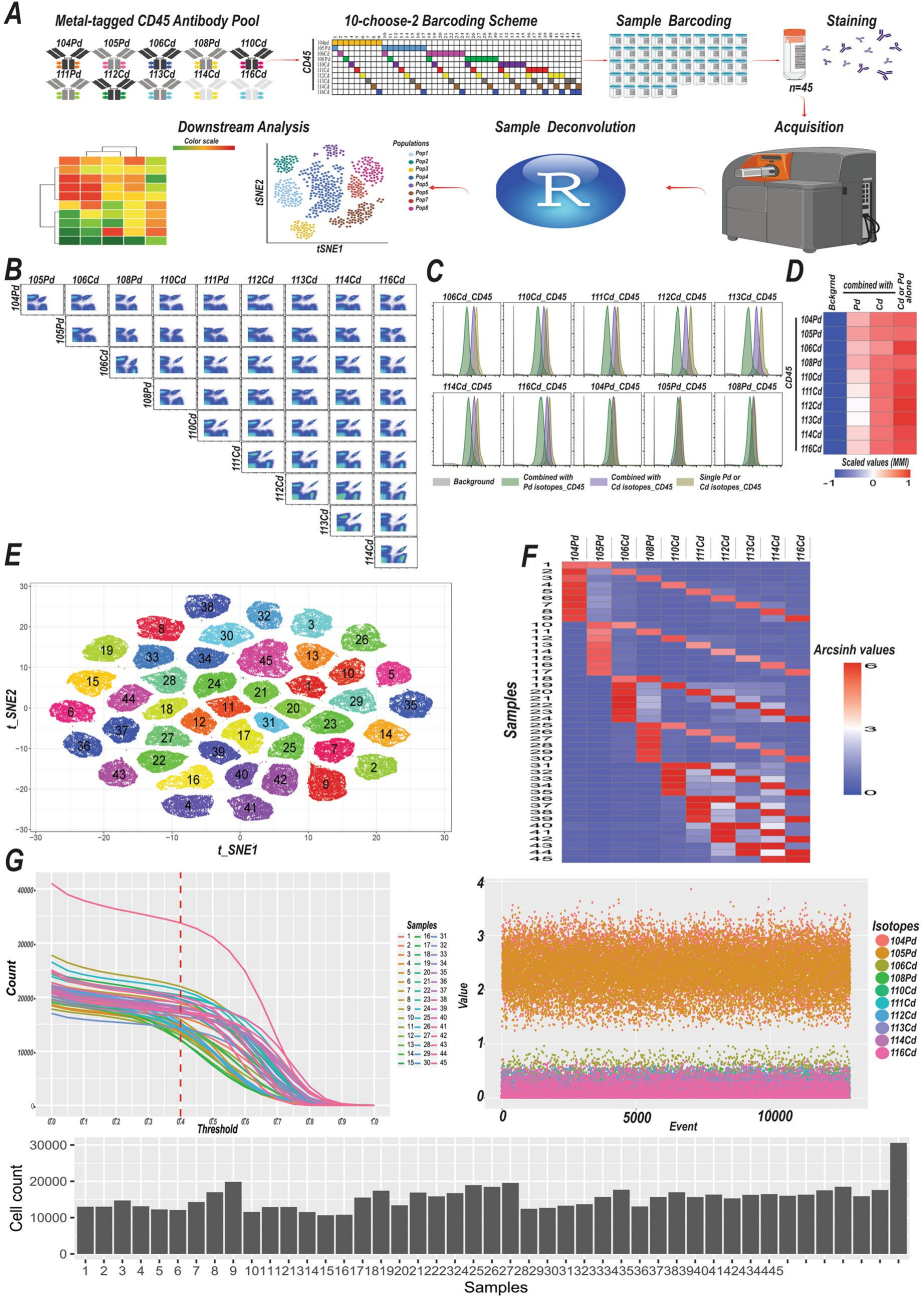
Live-cell barcoding platform utilizing MCP9-loaded Pd and Cd isotopes expands barcoding scheme. (**A**) Schematic representation of barcoding scheme (Created with BioRender.com). Samples were individually barcoded with a unique MCB composed of two different Pd or Cd-tagged CD45 antibodies. The pooled samples were processed and acquired on CyTOF machine simultaneously. Samples were deconvoluted to their identity using Premessa R package. Deconvoluted samples were then subjected to downstream analysis. (**B**) 10-choose-2 scheme is utilized to barcode 45 different experimental conditions. PBMCs from a healthy donor were labeled with 45 unique dual combinations generated from a pool of 10 Pd and Cd tagged CD45 antibodies. Separately labeled PBMCs were pooled together after labeling and run on a Helios CyTOF. Biaxial plots illustrate all possible dual combinations and signal intensities. (**C**) Signal intensities of Pd or Cd tagged CD45 antibodies are shown when Cd or Pd-tagged CD45 antibodies are used alone or in dual combinations with either Cd or Pd-tagged CD45 antibodies to label PBMCs. PBMCs were labeled with 45 MCBs and then pooled and acquired simultaneously. Positive events for each MCBs were selected. Cd or Pd positive events are gated out to calculate signal intensities in dual combinations with Pd and Cd isotopes, respectively. (**D**) The heatmap shows the mean signal intensities for 10 MCBs in (**C**). Values are scaled per row (**E**) Two-dimensional t-SNE plot shows pooled 45 samples in (**B**) on high-dimensional plane. (**F**) Heatmap shows the arcsinh-transformed mean signal intensities of CD45 antibodies tagged to 10 different isotopes used to barcode 45 samples in (**E**). (**G**) Pooled sampled are deconvoluted using Premessa R package.

We generated 10 different CD45-based barcoding reagents recognizing the same antigenic epitope. Next, we aimed to test various barcoding schemes for multiplexing in CyTOF. Combined use of multiple tags to barcode a single sample decreases the signal intensity for any given single tag due to competition for the same antigenic epitope, which could compromise signal resolution. Hence, we next assessed and compared staining indices by labeling samples with single, double, or triple combinations of five different Cd-tagged CD45 antibodies. Use of double or triple Cd-CD45 tags per individual sample was associated with a gradual decrease in the staining index (Fig. S3A). In addition, CD45 expression is variable across different leukocyte subsets^14^. To maintain acceptable signal resolution, reduce antibody consumption and achieve higher yields after sample deconvolution we therefore decided to use two mass-tag cell barcodes (MCBs) per sample. This led us to utilize a 10-choose-2 barcoding scheme, enabling us barcoding up to 45 experimental conditions (Fig. 3A). To test this barcoding scheme, we generated 45 dual antibody combinations out of a pool of 10 Pd- and Cd-tagged CD45 antibodies and stained PBMCs from a single healthy donor with 45 MCBs, permitting the assessment signal intensities of different dual combinations (Fig. 3B).

Interestingly, combining any Cd-tagged CD45 with Pd-tagged CD45 resulted in marked signal reduction while we observed relatively higher signal intensities if the Cd-tagged CD45 antibodies were used together with CD45-tagged with different Cd isotopes (Fig. 3C). Conversely, combining Pd-tagged CD45 with either Pd- or Cd-tagged CD45 resulted in modest signal shifts (Fig. 3C,D). However, despite a reduction in signal intensities with dual staining, each barcoded sample was distinctly represented when we assessed assigned barcoding tags using biaxial plots (Fig. 3B). Next, to assess the separation among all barcoded samples on a high dimensional plane, we generated two-dimensional t-SNE (t-distributed stochastic neighbor embedding) maps and utilized PhenoGraph to partition the barcoded dataset into subpopulations. Barcoded samples were situated apart from each other and PhenoGraph identified 45 distinct subpopulations (Fig. 3E). Each sample exhibited a staining profile compatible with the assigned barcodes (Fig. 3F, Fig. S3B).

Having established that this extended barcoding scheme using Pd- and Cd-tagged CD45 antibodies enabled pooling of 45 samples with convincingly distinct barcode separation on a high-dimensional plane, we next proceeded to deconvolute samples to their initial identity using Premessa R-package (Fig. 3G). As expected, deconvoluted samples displayed staining profiles compatible with their assigned barcodes (Fig. S3C). We achieved 93% yield with applied filters (minimum barcode separation: 0.4, and Mahalanobis distance: 30)^18^. We also performed tSNE and PhenoGraph analyses on 45 de-barcoded samples to assess sample purity. The samples were examined to determine if they contained any cells from the bulk samples to assess purity. We observed that each sample was composed of cells having unique assigned barcodes (Fig. S3D). Altogether, this live-cell barcoding platform offers an attractive and feasible approach that could significantly contribute to the field of CyTOF by significantly augmenting sample throughput.

### Utilizing the live-cell barcoding approach to interrogate T-cell populations in healthy individuals

**Figure 4:**
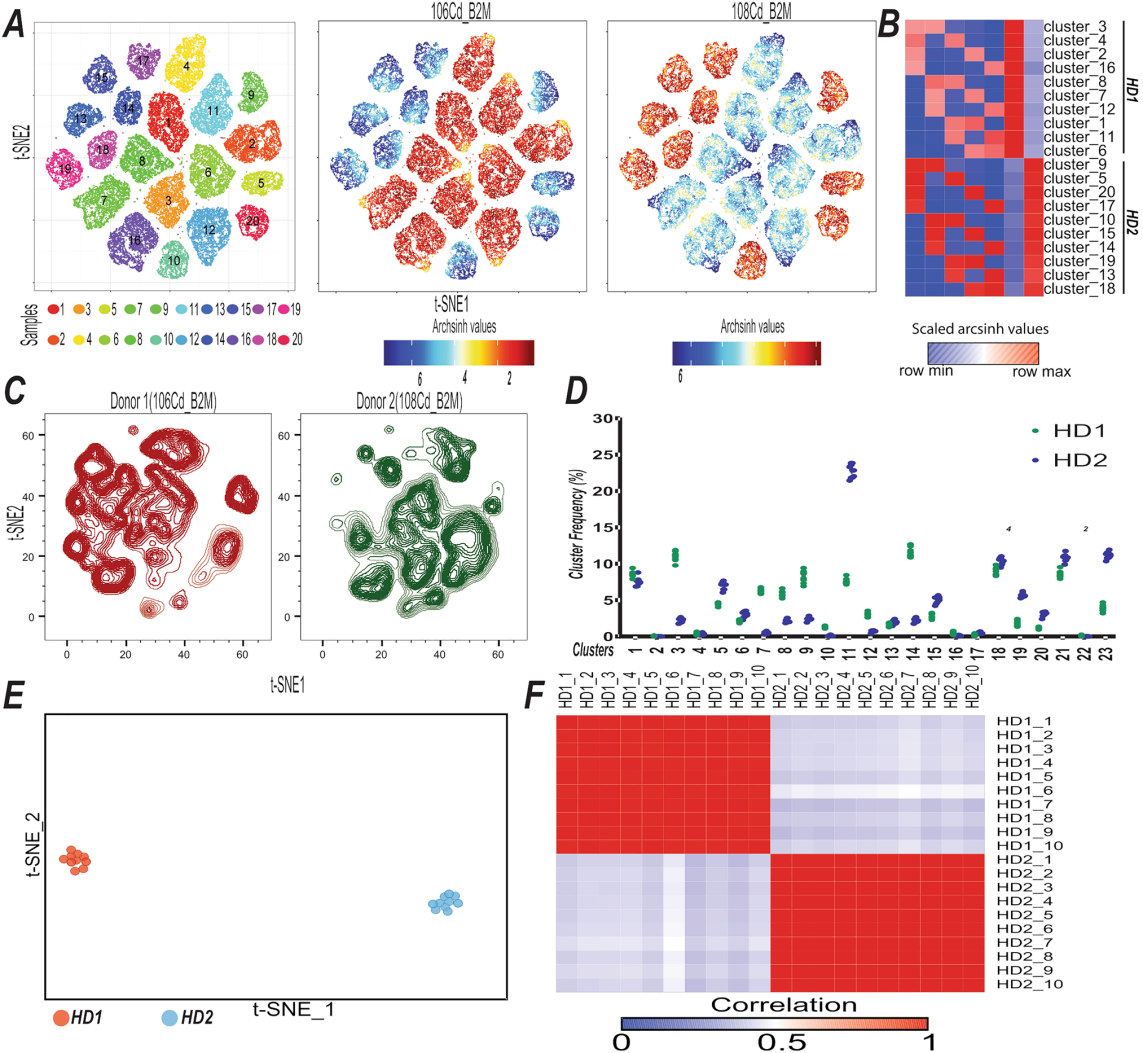
Live-cell barcoding facilitates assessment of T-cell compartment. (**A**) Two-dimensional t-SNE maps of pooled samples are shown and FlowSOM is used to identify the clusters in the dataset. PBMCs from two HDs were labeled with 106Cd_B2M and 108Pd_B2M antibodies separately and pooled. Five antibodies (110Cd, 111Cd, 112Cd, 114Cd, and 116Cd tagged to CD45) are used to generate 10 MCBs utilizing 5-choose-2 barcoding scheme. Pooled PBMC from two HDs were subjected to a second round of barcoding using 10 MCBs. t-SNE maps are colored for 106Cd_B2M (middle panel, HD1) and 108Pd_B2M (left panel, HD2). (**B**) Heatmap shows scaled values for clusters seen in (**A**). (**C**) Deconvoluted samples (n = 20) were subjected to t-SNE algorithm using all the parameters except barcoding tags for dimension reduction. Contour t-SNE plots for an equal number of cells show immune landscape of HD1 (106Cd_B2M) and HD2 (108Pd_B2M). (**D**) Clusters frequencies identified across two HDs using PhenoGraph algorithm (left). (**E**) PhenoGraph clusters of deconvoluted samples (n = 20) from two HDs are plotted on a t-SNE analysis (Right) Each dot represents a single sample. (**F**) Heatmap shows correlation among deconvoluted samples (Figure S4C) based on PhenoGraph cluster frequencies in (**D**).

Ultimately, we wanted to evaluate the utility of live-cell barcoding using this novel, extended CD45-based approach. To this end, we used frozen PBMCs isolated from two healthy donors (HDs). Other antibodies against abundantly expressed antigens could be utilized for live-cell barcoding and we also tagged B2M and CD298 antibodies with Cd and Pd isotopes and explored the utility of using these antibodies for live-cell barcoding. We observed that antigen density of B2M on PBMCs was comparable to CD45 while CD298 was less abundantly expressed (Figure S4A). First, we labeled PBMCs from two HDs with two different barcoding tags, 106Cd-B2M and 108Pd-B2M and pooled the samples. As a proof-of-concept, we used dual combinations of five different barcoding reagents that allowed barcoding for 10 different conditions. Pooled PBMCs from two HDs were split into 10 aliquots, and each labeled with a unique MCB. Barcoded samples were pooled and stained with a T-cell focused panel (Table S1). Dimension reduction and clustering using barcoding parameters identified 20 clusters (Fig. 4A), each having a phenotypic profile matching their assigned barcodes (Fig. 4B, Fig. S4B).

Next, we utilized Premessa algorithm to assign the barcoded cells to their initial identity (Fig. S4C). We then generated t-SNE maps using all deconvoluted samples and identified subpopulations in the dataset using PhenoGraph (Fig. S4D). In this simulative approach, we assumed that samples from the same donor would have the same cluster composition. We identified 23 distinct clusters from 20 samples (2750 cells/sample) from two HDs and evaluated their phenotypic profiles. PhenoGraph revealed the major immune subsets observed in these two HDs (Fig. S4D,E). Immune cluster composition differed between two HDs (Fig. 4C), samples from the same donor had significantly similar cluster composition (Fig. 4D) and there was significant similarity among subsamples from the same donor with regards to cluster frequencies (Fig. 4E,F), reflecting that samples were successfully allocated back to their original identity using this extended CD45-based barcoding approach and subsequent sample deconvolution. This small-scale approach proved the utility of this platform for multiplexing CyTOF and can significantly streamline CyTOF experimentation. Altogether, these findings demonstrate the utility of this novel live-cell barcoding approach using a pool of MCP9-loaded Pd and Cd-tagged CD45 antibodies. This approach substantially extends our barcoding capabilities, enables barcoding of significant number of samples, augments﻿﻿﻿﻿﻿﻿﻿﻿﻿﻿﻿﻿﻿﻿﻿﻿﻿ throughput, minimizes procedural variations, and enhances data quality.

## Discussion

CyTOF allows simultaneous interrogation of a wide array of cellular features including cell surface profiles^19^, cell cycle^20^, proliferation^21^, apoptosis^22^, metabolism^23^, phosphoproteins^24^, cytokine production^25^, transcription factors^26^ and multiplexed RNA profiles^27^ at single-cell resolution. Antibody staining variance, pipetting errors, detector drift between different samples on the same day and day-to-day variability of instrument performance are the main sources of technical variation. As a result, sample multiplexing has emerged as an invaluable strategy in the application of CyTOF by reducing tube-to-tube variation, eliminating sample carry-over, decreasing antibody consumption and enhancing throughput^7^. Generally, barcoding approaches have relied on the use of metal tags residing below the lanthanide mass range, thereby not impinging on lanthanide detection^7,8,28^. Intracellular barcoding using Pd isotopes is well-suited for interrogation of signaling pathways wherein a limited range of phenotypic profiling is sufficient to define dominant phenotypes in an experimental setting^29,30^. However, assessment of surface phenotype in fixed and permeabilized cells most likely does not reflect actual phenotypic profile. First, fixation/permeabilization alters surface antigens by introducing conformational changes of the proteins and irreversibly modifying antigenic epitopes recognized by antibodies. Second, some surface antigens are also localized within intracellular granules^31,32^.

To meet the growing demand for sample multiplexing in this field while preserving surface epitopes, efforts were made towards developing innovative surface barcoding approaches. The attractive approach has been to explore metals other than lanthanides including Pd^5,12,16^, platinum^8,10,14^, tellurium^13^, indium^14,16,33^ and yttrium^33^ for live-cell barcoding. Bifunctional chelating agents, ITCBE and mDOTA, were used to capture 6 Pd isotopes and generate MCBs for live-cell barcoding. However, the main limitation with Pd-based barcoding is that Pd isotopes elicit weaker signals. Therefore, attempts have been made towards combined use of 5 Pd isotopes, other than 102Pd due to its high cost, with other low sensitivity metals, such as indium isotopes, for sample multiplexing^16^. Weaker signals associated with Pd isotopes are partly related to use of monomer chelators capable of capturing limited number of metals compared to polymeric chelators^5^. Development of novel Pd-specific chelators could significantly improve signal-to-noise ratio. Use of MCP9 polymers specifically to chelate Cd isotopes is an exemplary condition, illustrating how novel chelators could improve signal intensities of low sensitivity metals. Cd metals are situated at the lower end of the sensitivity spectrum and are recommended to be paired with abundant antigens, although MCP9 polymers can capture higher numbers of metal isotopes. We reasoned that MCP9 loaded with different Cd and Pd isotopes could be paired with CD45 to generate unique MCBs. As a proof-of-concept, we chose to generate CD45-based MCBs to barcode PBMCs as CD45 is ubiquitously expressed on leukocytes although with varied expression across different subsets^34^. The live-cell barcoding scheme that we proposed in this study includes 10 mass tags, seven Cd and three Pd, and enabled us to effectively pool up to 45 samples using a 10-choose-2 scheme. This significantly expands the number of samples that can be barcoded using a live-cell barcoding approach in comparison to previous studies utilizing live-cell barcoding approaches^16^. This approach can be further extended to generate barcoding reagents by pairing MCP9/Cd isotopes with ubiquitously expressed markers as described previously^12,23^, thereby permitting to barcode different cell types other than hematopoietic cells. In addition, this approach could further be tailored to meet unique experimental needs. For examples seven barcodes could be used to barcode 21 different samples and the remaining three Cd, preferably the Cd channels receiving less spillover from neighboring channels, can be paired with abundant antigens to increase the number of assessed analytes. On the other hand, barcoding scheme could be further extended by utilizing CD45 antibodies paired with yttrium-89 and/or 102Pd together with 10 MCBs that we generated in order to barcode a far greater number of experimental conditions. Utilizing 11-choose-2 or 12-choose-2 barcoding scheme can allow to pool up to 55 or 66 samples, respectively.

We initially conjugated seven Cd isotopes loaded to MCP9 polymer to CD45 (HI30). Having two overlapping isotopes with Pd (106Pd and 110Pd) enabled us comparing signal intensities and metal content of similar mass weight, Cd-loaded MCP9 polymer versus Pd-loaded mDOTA monomer tagged with CD45. Antibody capture bead approach demonstrated that the number of metals on MCP9 polymer was significantly higher compared to Pd loaded to mDOTA polymers (Fig. 2B). Thus, the availability of Cd/MCP9 for antibody tagging not only enriched the barcoding toolbox but also provided a means to generate superior signal intensities using low-sensitivity metals. Using three MCBs per sample out of seven could be tolerable, enabling multiplexing up to 35 samples. However, using three MCBs per sample was associated with lower staining index (Fig. S3A) and could possibly compromise total yield after sample deconvolution. Therefore, we adopted a two-tag per-sample approach to maintain favorable signal resolution and asked the question if we could incorporate three Pd-tagged antibodies (104Pd, 105Pd, and 108Pd) to Cd-based barcoding scheme, allowing us to use 10 unique mass tags. Rather than using ITCBE and mDOTA monomers for Pd chelation we demonstrated that MCP9 can also capture bivalent Pd metals (Fig. 2A). This approach offers great advantage over previously described antibody conjugation approaches using Pd isotopes loaded either to ITCBE or mDOTA^5^. ITCBE is not readily dissolvable in “conjugation-friendly” buffers. mDOTA is another chelator that could be utilized to capture bi- and trivalent metals, and readily dissolves in H_2_O. However, aqueous mDOTA is not stable and the user should proceed to Pd loading to mDOTA immediately and possibly cannot reuse dissolved mDOTA. On the other hand, using MCP9 polymers for Pd loading significantly simplifies and shortens antibody tagging using Pd isotopes. We showed that antibody tagging using Pd loaded MCP9 generated favorable signal intensities, albeit lower than Cd-tagged antibodies, suggesting that MCP9 may have higher affinity for Cd than Pd isotopes (Fig. 2B). However, to load Pd metals to MCP9 we used three Pd isotopes (104Pd, 105Pd, and 108Pd) that were already dissolved in 5 N HCl (hydrochloric acid) as they were previously used for ITCBE and mDOTA loading. Optimal pH is essential for metal chelation and Pd(NO_3_)_2_ is readily soluble in 100 mM 4-(2-hydroxyethyl)-1-piperazineethanesulfonic acid^6^. Use of enriched Pd isotopes dissolved in 4-(2-hydroxyethyl)-1-piperazineethanesulfonic acid or H2O to load onto MCP polymer may further improve chelation and augment signal intensities for Pd-MCP9 tagged CD45 antibodies.

Altogether, we describe a novel live-cell barcoding scheme based on combined use of Cd and Pd isotopes paired with CD45, enabling the pooling of significantly higher number of samples with preserved superior signal-to-noise ratios. Importantly, this approach can be tailored to meet specific experimental needs, either through incorporating more mass tags residing at the lower end of sensitivity spectrum to extend barcoding depth or using a certain of number MCBs to barcode a relatively lower number of samples while using the remaining isotopes to assess more analytes. Of note, sample preparation and acquisition in CyTOF leads to significant cell loss. Therefore, starting with at least a million cells is required for separately processed and acquired samples. This brings up a challenge in such settings where high cell numbers are not obtainable. Hence, samples with very low cell counts could be pooled, processed and acquired together using this live-cell barcoding platform. Performing intracellular barcoding after surface staining and fixation/permeabilization is not suitable for samples with low number of cells as a significant portion of samples is lost during sample processing. On the other hand, surface barcoding is well-suited to combine small samples into a larger pool, thus enabling the achievement of acceptable cell recovery after sample processing, acquisition and deconvolution. This approach could be particularly useful for the in-depth interrogation of tumor cells and their microenvironment in patients with hypocellular bone marrows after treatment of hematological malignancies. Mapping the recovering immune system and residual tumor cells, termed minimal residual disease or MRD, is challenging due to therapy-related excessively low cell counts. Consequently, using this platform in settings wherein sample cell counts are low can overcome this sort of challenges and provide significant benefit to clinical research.

## Methods

### Sample preparation

Peripheral blood samples were obtained from healthy donors after obtaining written informed consent in accordance with The University of Texas MD Anderson Cancer Center Institutional Review Board (IRB) guidelines. All the samples in this study were deemed human subject research exempt and the procedures were approved by The University of Texas MD Anderson Cancer Center IRB (protocol number LAB02-395).

PBMCs were isolated by density-gradient centrifugation using lymphocyte separation medium (Lymphoprep; Axis Shield, Oslo, Norway). Isolated PBMCs were cryopreserved in freezing media containing 10% dimethyl sulfoxide and 90% fetal bovine serum. Frozen PBMCs were thawed rapidly in 37^o^ C water bath and half-thawed samples were transferred to pre-warmed culture media (90% RPMI 1640) containing 2 mM GlutaMAX TM-I, 1% Penicillin–Streptomycin and 10% fetal bovine serum) and supplemented with 50 IU-ml benzonase (Sigma-Aldrich, St. Louis, MO). Thawed PBMCs were washed once and incubated overnight at 37° C with air containing 5% CO_2_ before staining.

### Antibody conjugation

Antibodies and corresponding metal tags are detailed in Table S1. Unlabeled antibodies were purchased in carrier-free form and conjugated to lanthanides and indium isotopes using Maxpar X8 polymer per manufacturer’s instructions (Fluidigm, San Francisco, CA). Lanthanides and Indium isotopes were procured from Fluidigm and Traces Sciences, respectively. Monoisotopic Indium salts were dissolved in distilled H2O to 1 M stock solution, then further diluted to 50 mM in L buffer (Fluidigm, San Francisco, CA) and loaded onto Maxpar X8 polymer (Fluidigm, San Francisco, CA). As for conjugation of unlabeled antibodies to monoisotopic cisplatin containing 194Pt or 198Pt isotopes, we used a protocol adapted from a previously published study^9^. Briefly, 100 µg of unlabeled carrier-free antibody was washed with R buffer in a 50 kilodalton (kDa) spin filter column (EMD Millipore), reduced in R buffer using 4 mM final concentration of TCEP for 30 min at 37^o^ C and then washed twice with C buffer. The reduced antibody was resuspended in C buffer, monoisotopic cisplatin compounds containing either 194Pt or 198Pt (Fluidigm, San Francisco, CA) was added to a final concentration of 100 µM and total volume was adjusted to 400 µl with C buffer. The antibody-cisplatin mixture was incubated for two hours at 37^o^ C and washed four times with W buffer (Fluidigm, San Francisco, CA). Antibody concentration was determined by absorbance reading 280 nm using Nanodrop 2000 (Thermo Fisher Scientific, Waltham, MA) All conjugated antibodies were then diluted to 0.5 mg/ml final concentration either in PBS-based antibody stabilization solution or Low Cross-Buffer (Candor Bioscience GmbH, Wangen, Germany) supplemented with 0.05% sodium azide (Sigma-Aldrich, St. Louis, MO). Serial titration experiments were performed to determine the concentration giving the optimal signal-to-noise ratio for each antibody.

### Generation of Cd and Pd barcoding reagents

Seven Cd isotopes were acquired from Fluidigm and were conjugated to CD45 antibody (Clone HI30; Biolegend, San Diego, CA) using Maxpar MCP9 antibody labeling kit (Fluidigm, San Diego, CA) per manufacturer’s instructions. Briefly, 13 µl of Cd isotopes were loaded onto 200 µg of MCP polymer suspended in 87 µl of L buffer and incubated for one h at 37 °C in water bath. Following incubation, Cd loaded MCP9 polymer was washed twice in L and once in C buffer on 3 kDa spin filter and suspended in 60 µl C-buffer. 100 µg of CD45 or B2M antibodies was washed twice in R buffer and then reduced in 4 mM TCEP in 100 µl R buffer for 30 min at 37 °C. The reduced antibody was then washed twice in C-buffer. Cd-loaded MCP polymer was then transferred to the 50 kDa filter containing the reduced antibody. Reduced antibody/Cd-loaded polymer mixture was incubated for 90 min at 37 °C. After incubation, antibodies were transferred to 100 kDa filter and washed four times for five min at 5000 × *g*. Antibody concentration was determined based on absorption at 280 nm using Nanodrop 2000 (Thermo Fisher Scientific, Waltham, MA) All conjugated antibodies were then diluted to 0.5 mg/ml final concentration in HRP-protector (Candor Bioscience GmbH, Wangen, Germany).

Five monoisotopic palladium nitrate compounds, 104Pd, 105Pd, 106Pd, 108Pd, and 110Pd, were sourced from Trace Sciences and dissolved in hydrochloric acid (HCl) to 50 mM concentration. We adapted a protocol previously published by Han et al*.*^5^ and used Maleimido-mono-amide-DOTA (mDOTA, Macrocyclics, Dallas, TX) to chelate Pd isotopes. Briefly, mDOTA was dissolved in double-distilled water (ddH2O) at 25 mM concentration. Palladium isotopes were mixed with mDOTA at 1:1.2 molar ratio. The mixture was immediately snap-frozen and lyophilized overnight. Lyophilized Pd loaded mDOTA complex was dissolved in dimethyl sulfoxide to 10 mM concentration. One microliter of Pd-loaded DOTA was added to partially reduced antibody suspended in 100 µl C-buffer. The mixture was then incubated for at least one h at 37 °C. Zeba 7 kDA spin columns (Thermofisher, USA) were used for antibody purification. Briefly, Zeba 7 kDA spin columns were spun to remove storage solution and then washed twice with 300 µl W buffer (Fluidigm). Pd-loaded mDOTA and antibody mixture was transferred to Zeba 7 kDA spin column and spun at 1,500 × *g* for two min. The antibody concentration was measured based on absorption readout at 280 nm. For solvent removal before suspending in antibody stabilizing solution the flow-through was then transferred to a new 50 kDa spin filter and spun at 12,000 × *g* for five min. Antibodies tagged with Pd-loaded mDOTA was diluted to 0.5 mg/ml in PBS-based antibody stabilization solution or LowCross-Buffer (Candor Bioscience GmbH, Wangen, Germany) supplemented with 0.05% sodium azide (Sigma-Aldrich, St. Louis, MO).

### Antibody conjugation using Pd-loaded MCP9 polymers

Two Pd isotopes, 106Pd and 110Pd, overlap with 2 Cd isotopes, 106Cd and 110Cd, having similar mass weights. Hence, three monoisotopic palladium nitrate compounds, 104Pd, 105Pd and 108Pd, were previously dissolved in HCl to 50 mM concentration to load onto DOTA and ITCBE chelators in accordance with earlier reports^5^. Pd isotopes suspended in 5 N HCl were lyophilized overnight and suspended in nitric acid to generate Pd(NO_3_)_2_ salts. Isotopically enriched Pd nitrate solution was lyophilized overnight and suspended in water to 50 mM concentration. We utilized a similar approach for Cd-MCP9 antibody conjugation protocol to load Pd metals onto MCP9 polymers. Briefly, 13 µl of monoisotopic Pd isotope was loaded onto 200 µg of MCP polymer and washed twice with L and once with C buffer after incubation at 37 °C for one hour. Pd-loaded MCP9 polymer was then mixed with reduced CD45 antibody and incubated for 90 min at 37 °C. After incubation, antibodies were transferred to 100 kDa filter and washed using W buffer (Fluidigm, San Diego, CA). Antibody concentration was determined based on absorption at 280 nm using Nanodrop 2000 (Thermo Fisher Scientific, Waltham, MA) All conjugated antibodies were then diluted to 0.5 mg/ml final concentration in HRP-protector (Candor Bioscience GmbH, Wangen, Germany).

### Capture bead labeling

Anti-mouse Ig kappa antibody capture beads (BD Biosciences, San Jose, CA) were used to assess and compare signal intensity for Cd and Pd labeled antibodies per a previously published protocol^35^. Briefly, equal amounts of antibody capture beads were stained with Cd and Pd-labeled antibodies and incubated at room temperature for 20 min. Following incubation, capture beads were washed twice with 0.5% bovine serum albumin (BSA)/PBS solution (staining buffer) and then fixed using 1.6% paraformaldehyde (PFA) (Electron Microscopy Sciences, Hatfield, PA) for one hour at room temperature (RT). Fixed beads were washed twice with 0.5% BSA/PBS and twice with ddH_2_O. The beads labeled with single Cd or Pd-tagged CD45 antibody were suspended in 500 µl of ddH2O and acquired individually on a Helios mass cytometer (Fluidigm, San Diego, CA). Single events were chosen following gating on event length and Cd or Pd channels of interest using FlowJo version 10.6 (TreeStar; Ashland, OR).

### Live-cell barcoding

PBMCs including up to 2 × 10^6^ cells were stained with different combinations of Cd (i.e., 106Cd, 110Cd, 111Cd, 112Cd, 113Cd, 114Cd, and 116Cd) and Pd (i.e., 104Pd, 105Pd, and 108Pd) tagged CD45 antibodies at a final concentration of 2.5 µg/ml per antibody and incubated at RT for 30 min. We utilized a 10-choose-2 barcoding scheme allowing us to barcode up to 45 different experimental conditions with doublet filtering scheme. Samples, each tagged with a unique barcode, were washed three times with staining buffer. Washed samples were then pooled and incubated with 5uM of natural abundance cisplatin (Enzo, Farmingdale, NY) or 1uM of monoisotopic cisplatin-196Pt (Neonest AB, Stockholm, Sweden) for one min at RT.

### Sample staining

Pooled samples were blocked with 5 µl of human Trustain FcX (Biolegend, San Diego, CA), Fc receptor blocking solution, for 10 min at RT. Cells were then stained with a collection of T-cell focused antibodies and incubated 30 min at RT. The antibody panel is presented in Table S1. Cells were washed twice with staining buffer after incubation, fixed in 1.6% PFA for 10 min at RT, permeabilized in 90% methanol at − 20 °C for 60 min. Permeabilized cells were washed twice with staining buffer and stained with intracellular antibodies. Cells were washed twice with staining buffer following incubation for 30 min at 4 °C, resuspended in intercalator solution (1.6% PFA in PBS with 125 nM iridium nucleic acid intercalator) and incubated at 4 °C overnight.

### Sample acquisition and data processing

Samples were washed twice with cell staining buffer, resuspended in 1 ml of MilliQ dH_2_O, filtered through 35 µm nylon mesh (cell strainer cap tubes, BD, San Jose, CA), counted and washed before sample introduction. Samples were suspended at a concentration of 0.6 × 106/ml in MilliQ ddH_2_O supplemented with 10% EQ Four Element Calibration Beads (Fluidigm, San Francisco, CA) and acquired at 300 events/second on Helios instrument using CyTOF Software version 6.7.1016 (Fluidigm, San Francisco, CA). CyTOF data were normalized based on signal drift over time using CyTOF Software.

### De-barcoding and data analysis

Deconvolution of pooled sample sets was performed using Premessa R-package. (https://github.com/ParkerICI/premessa). De-barcoding parameters were adjusted to allow optimal barcode separation. Initial data processing of deconvoluted samples was performed by using Flowjo version 10.6. Calibration beads were gated out and singlets were chosen based upon DNA content and event length. Dead cells were excluded by selecting cells with low cisplatin uptake. Desired populations of interest are gated on and then exported for downstream analyses. We used t-Distributed Stochastic Neighbor Embedding (t-SNE)^36^ and uniform manifold approximation and projection (UMAP)^37^ for dimension reduction, and FlowSOM^38^ and PhenoGraph clustering algorithms in Cytofkit^39^ using R software version 3.6.3 (R Core Team, 2020) for the data presented in Figs. 2B, 3E,G, 4A,C. R: a language environment for statistical computing. R Foundation for Statistical Computing, Vienna, Austria (URL https://www.R-project.org). The analyses were performed by using all the markers except for the parameters used for manual gating to identify and export downstream subpopulations.

## Supplementary Information

Supplementary Information.
