# Supplementary material for: Extended live-cell barcoding approach for multiplexed mass cytometry
Source: Sci Rep. 2021 Jun 11;11:12388. doi: 10.1038/s41598-021-91816-w (PMC8196040; doi:10.1038/s41598-021-91816-w)
Supplement: Supplementary file 1 — Supplementary Information. [file 41598_2021_91816_MOESM1_ESM.docx]

**Supplementary Data**

**Table S1.** List of antibodies used for multiplexed mass cytometry.

| **Isotope** | **Metal** | **Antigen** | **Clone** | **Polymer** | **Vendor** | **Category** |
| --- | --- | --- | --- | --- | --- | --- |
| 104 | Pd | CD45 | HI30 | MCP9/mDOTA | BioLegend | Barcoding |
| 105 | Pd | CD45 | HI30 | MCP9/mDOTA | BioLegend | Barcoding |
| 106 | Cd/Pd | CD45 | HI30 | MCP9/mDOTA | BioLegend | Barcoding |
| 108 | Pd | CD45 | HI30 | MCP9/mDOTA | BioLegend | Barcoding |
| 110 | Cd/Pd | CD45 | HI30 | MCP9/mDOTA | BioLegend | Barcoding |
| 111 | Cd | CD45 | HI30 | MCP9 | BioLegend | Barcoding |
| 112 | Cd | CD45 | HI30 | MCP9 | BioLegend | Barcoding |
| 113 | In/Cd | CD8/CD45 | RPA-T8/HI30 | mDTPA | BioLegend | Surface |
| 114 | Cd | CD45 | HI30 | MCP9 | BioLegend | Barcoding |
| 115 | In | CD3 | HI30 | mDTPA | BioLegend | Surface |
| 116 | Cd | CD45 | HI30 | MCP9 | BioLegend | Barcoding |
| 141 | Pr | CCR6 | G034E3 | mDTPA | Fluidigm | Surface |
| 142 | Nd | CD27 | M-T271 | mDTPA | BD | Surface |
| 143 | Nd | CD56 | NCAM16.2 | mDTPA | BD | Surface |
| 144 | Nd | CD26 | BA5b | mDTPA | BioLegend | Surface |
| 145 | Nd | NKG2A | REA110 | mDTPA | Miltenyi | Surface |
| 146 | Nd | CD49F | GoH3 | mDTPA | BD | Surface |
| 147 | Sm | CD127 | REA614 | mDTPA | Miltenyi | Surface |
| 148 | Nd | CCR2 | REA264 | mDTPA | Miltenyi | Surface |
| 149 | Sm | CCR4 | 205410 | mDTPA | R&D | Surface |
| 150 | Nd | CD28 | CD28.2 | mDTPA | BioLegend | Surface |
| 151 | Eu | Tbet | O4-46 | mDTPA | BD | Intracellular |
| 152 | Sm | TIGIT | MBSA43 | mDTPA | Thermofisher | Surface |
| 153 | Eu | CD45RA | HI100 | mDTPA | BioLegend | Surface |
| 154 | Sm | Ki-67 | 20Raj1 | mDTPA | Thermofisher | Intracellular |
| 155 | Gd | CCR7 | [G043H7](https://www.biolegend.com/en-us/search-results?Clone=G043H7) | mDTPA | BioLegend | Surface |
| 156 | Gd | TIM3 | [F38-2E2](https://www.biolegend.com/en-us/search-results?Clone=F38-2E2) | mDTPA | BioLegend | Surface |
| 157 | Gd | CD19 | HIB19 | mDTPA | BioLegend | Surface |
| 158 | Gd | 2B4 | C1.7 | mDTPA | BioLegend | Surface |
| 159 | Tb | ICOS | [C398.4A](https://www.biolegend.com/en-us/search-results?Clone=C398.4A) | mDTPA | BioLegend | Surface |
| 160 | Gd | PD-1 | EH12.2H7 | mDTPA | BD | Surface |
| 161 | Dy | CXCR3 | G025H7 | mDTPA | BioLegend | Surface |
| 162 | Dy | Eomes | WD1928 | mDTPA | Thermofisher | Intracellular |
| 163 | Dy | CD137 | 4B4-1 | mDTPA | BD | Surface |
| 164 | Dy | CTLA-4 | BN13 | mDTPA | Bioxcell | Surface |
| 165 | Ho | CD25 | 2A3 | mDTPA | BD | Surface |
| 166 | Er | LAG3 | REA351 | mDTPA | Miltenyi | Surface |
| 167 | Er | Perforin | B-D48 | mDTPA | Diaclone | Intracellular |
| 168 | Er | CD160 | 688327 | mDTPA | R&D | Surface |
| 169 | Tm | CD69 | FN50 | mDTPA | BD | Surface |
| 170 | Er | CD39 | A1 | mDTPA | Bio-Rad | Surface |
| 171 | Yb | CD161 | 191B8 | mDTPA | Miltenyi | Surface |
| 172 | Yb | KLRG1 | [13F12F2](https://www.thermofisher.com/antibody/product/53-9488-42) | mDTPA | Thermofisher | Surface |
| 173 | Yb | CD95 | DX2 | mDTPA | BD | Surface |
| 174 | Yb | GITR | DT5D3 | mDTPA | Miltenyi | Surface |
| 175 | Lu | CD4 | RPA-T4 | mDTPA | BioLegend | Surface |
| 176 | Yb | CD38 | REA572 | mDTPA | Miltenyi | Surface |
| 191 | Ir | DNA | N/A | N/A | Fluidigm | Other |
| 193 | Ir | DNA | N/A | N/A | Fluidigm | Other |
| 194 | Pt | CD57 | HCD57 | N/A | BioLegend | Surface |
| 195 | Pt | live-dead | N/A | N/A | Enzo | Other |
| 196 | Pt | live-dead | N/A | N/A | Enzo | Other |
| 198 | Pt | HLA-DR | L243 | N/A | BioLegend | Surface |
| 209 | Bi | CLA | HECA-452 | mDTPA | BD | Surface |

**
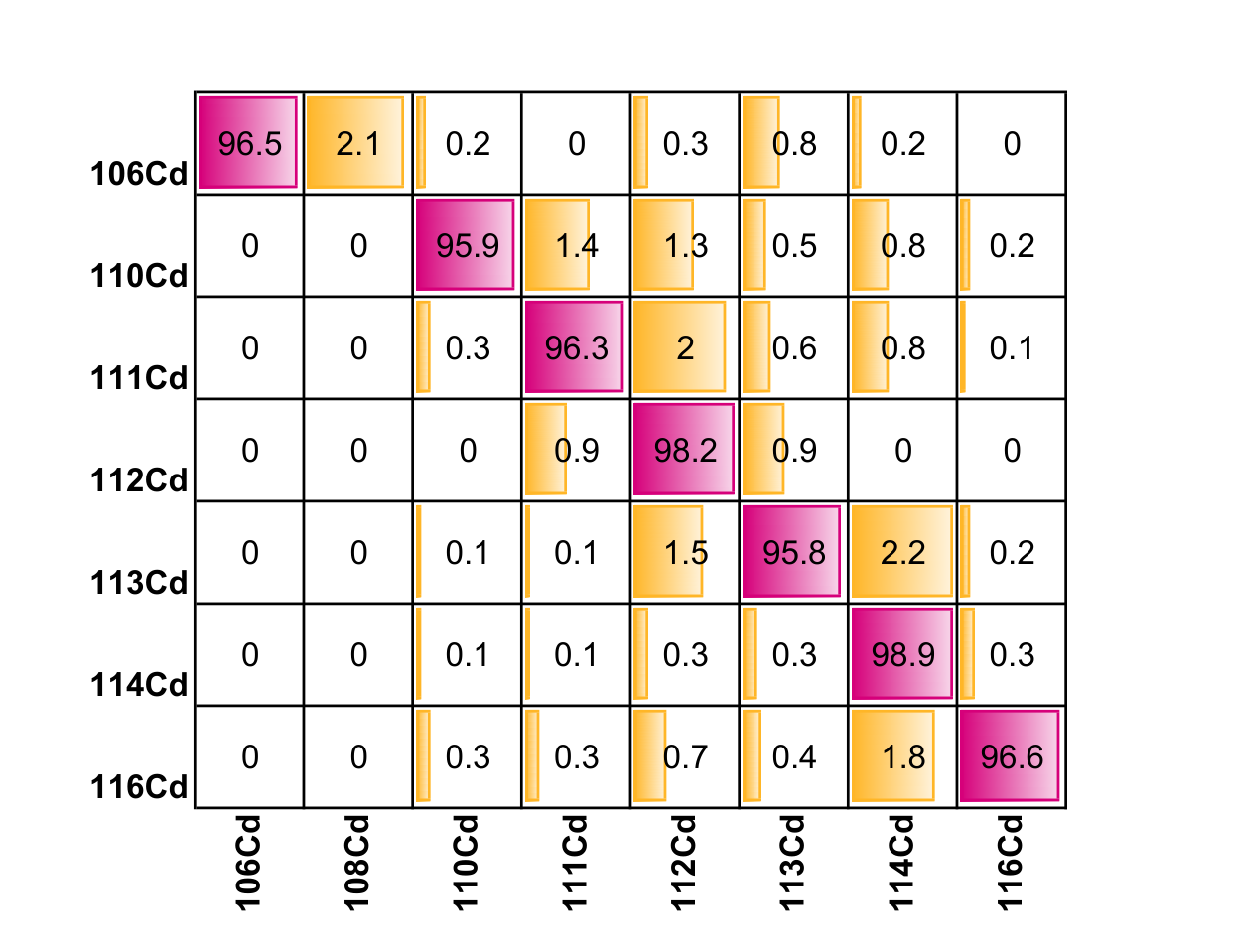

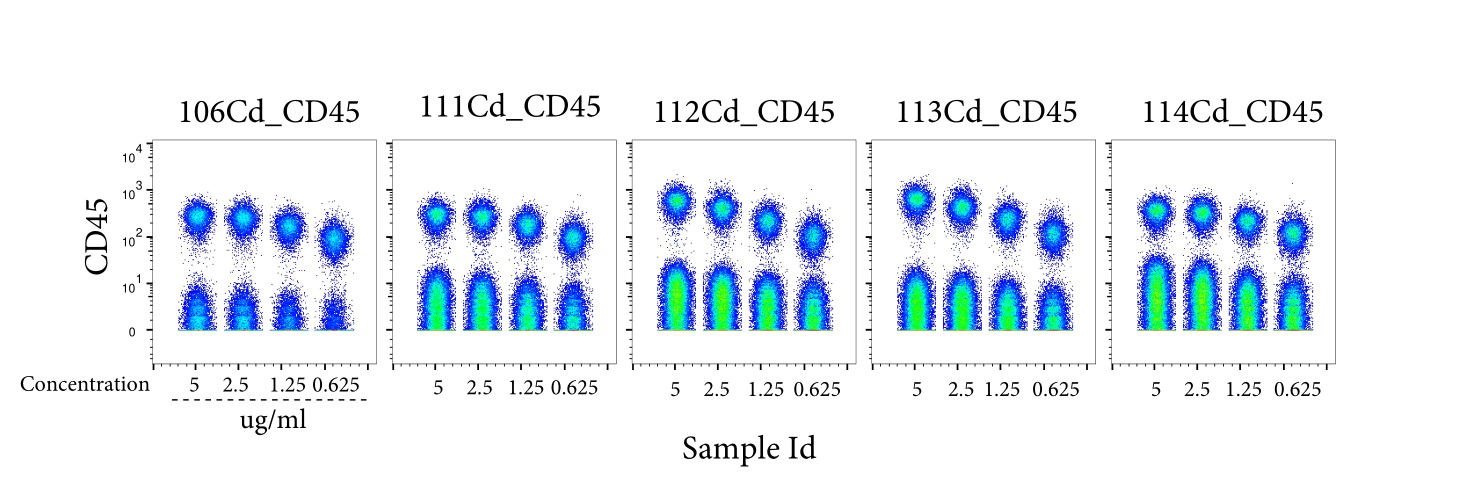
**

**Figure S1**

**Supplemental Figures**

**B**

**A**

**Supplementary** **Figure 1**. **Serial dilution and signal intensities of Cd-tagged CD45 antibodies**. (A) Data shown are concatenated FCS files showing serial dilution of CD45 antibodies tagged with 5 different Cd isotopes, 106Cd, 111Cd, 112Cd, 113Cd and 114Cd. (B) Purity matrix for 7 Cd isotopes (adapted from Fluidigm). The number in the cells show metal impurities for each cadmium isotope (row)

**
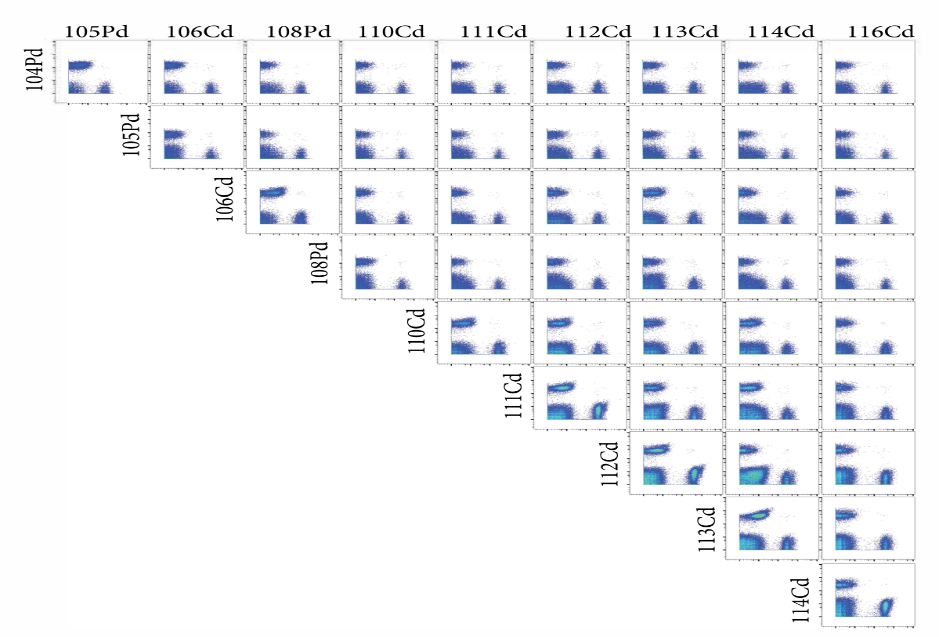

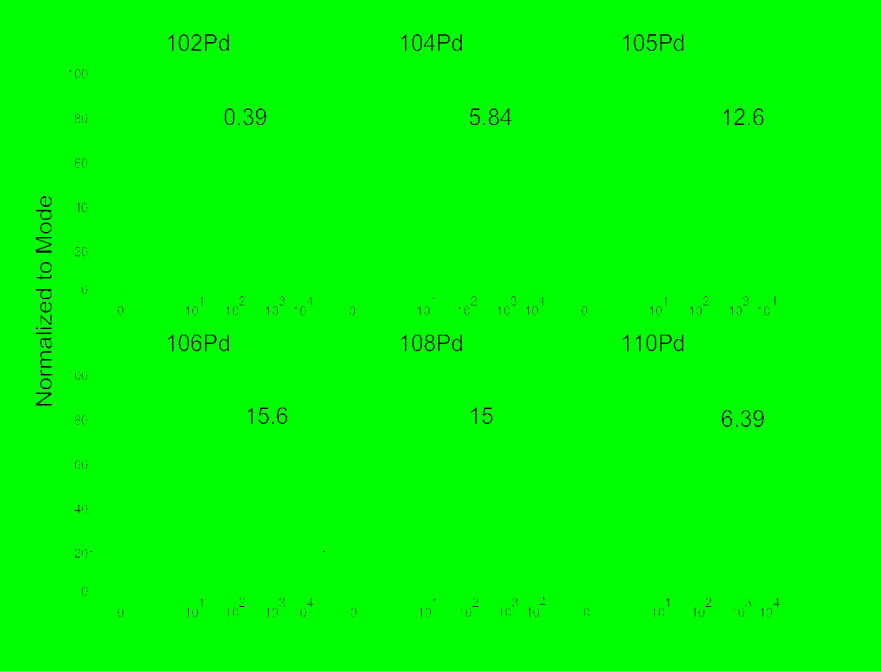
**

**A**

**B**

**Figure S2**

**C**

**
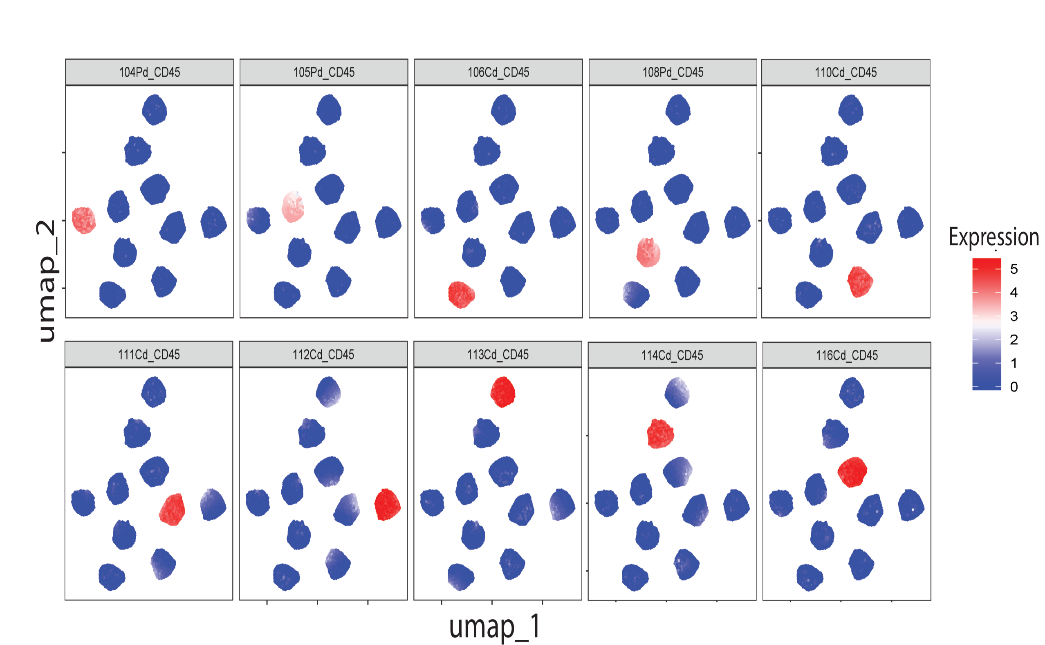
**

**Supplementary** **Figure 2. Evaluation of signal intensities of 10 MTBs generated using MCP9 polymer.**  (A) PBMCs are labeled with MCP9-loaded natural abundance Pd and signal intensities for 6 Pd isotopes, 102Pd (red), 104Pd (orange), 105Pd (yellow), 106Pd (green), 108Pd (blue) and 110Pd (purple) are shown as histograms. (**B**) PBMCs from a healthy donor were stained separately with 10 different MTBs, and then pooled and acquired simultaneously. The biaxial plots depict 45 different dual combinations observed when 10 tags were used. (C) Umap plots were generated and FlowSOM algorithm was implemented using expression levels for 10 different tags used for sample labeling. Umap plots were colored based on arcsinh values for each parameter utilized for dimension reduction and clustering.

**
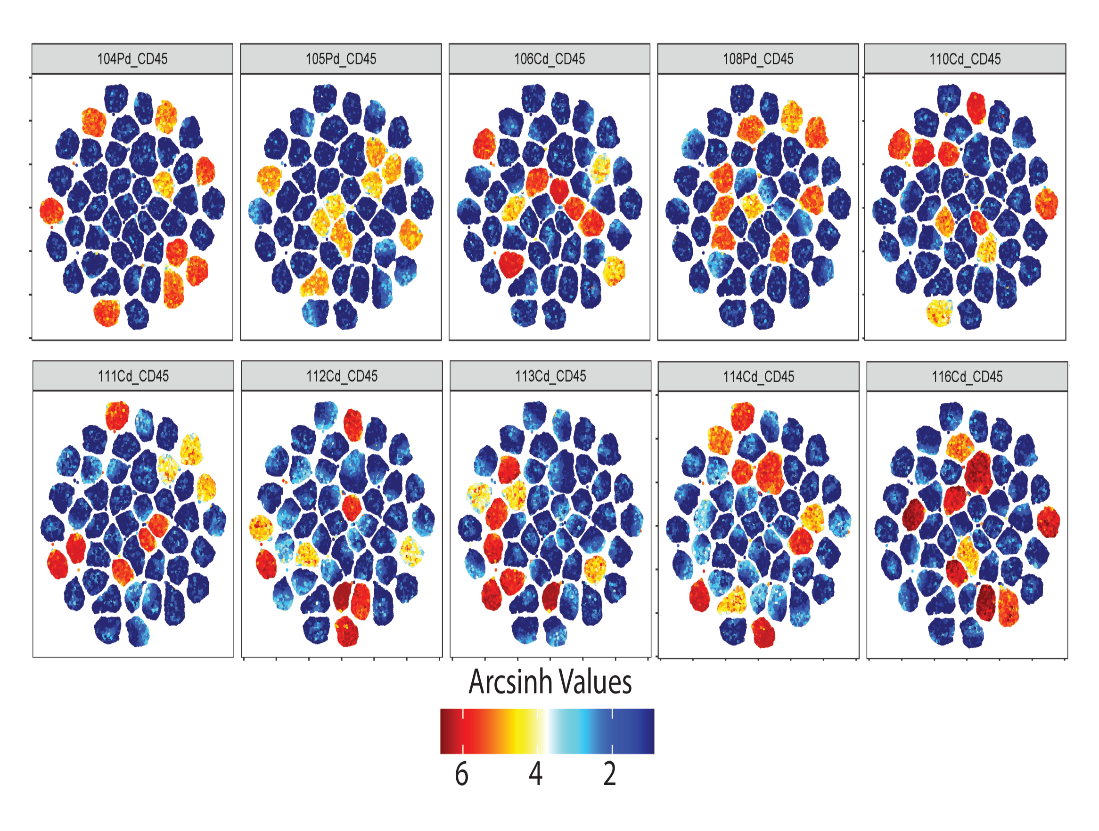

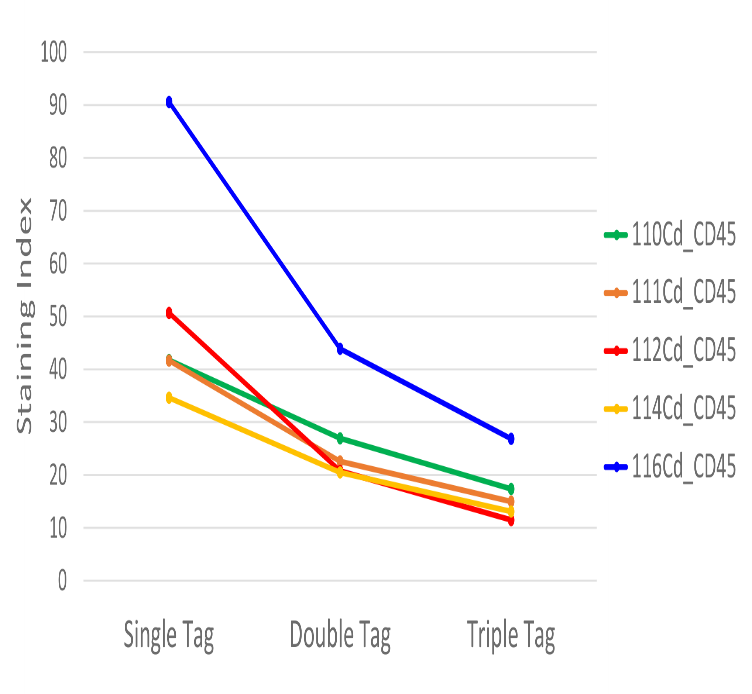
**

**Figure S3**

**B**

**A**

**D**

**C**

**
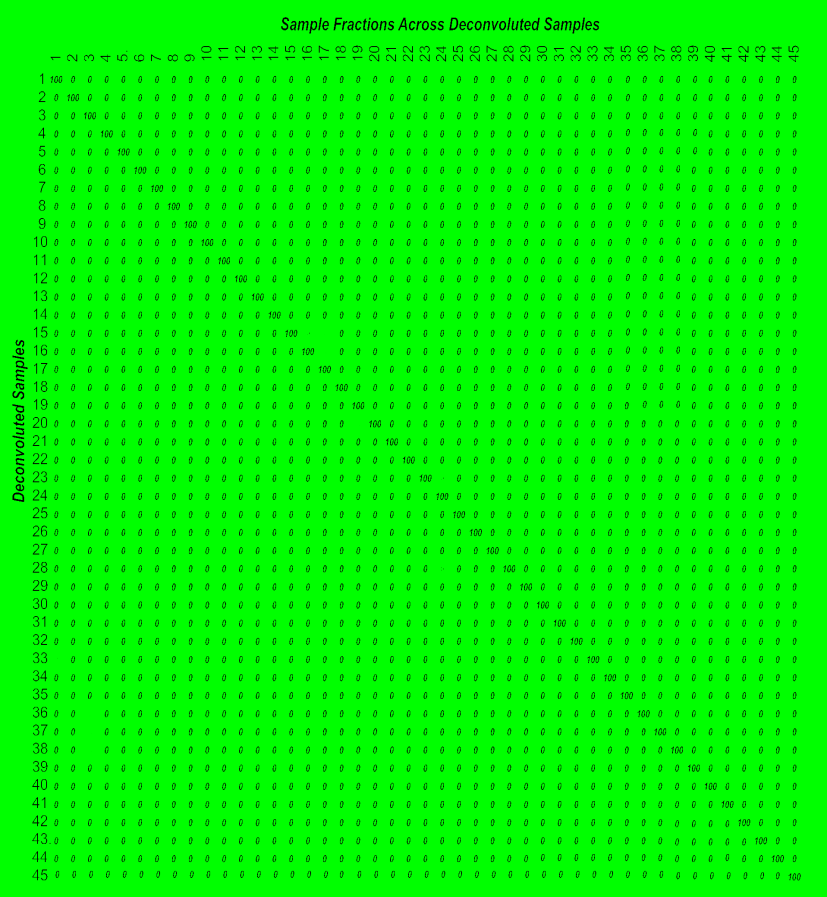

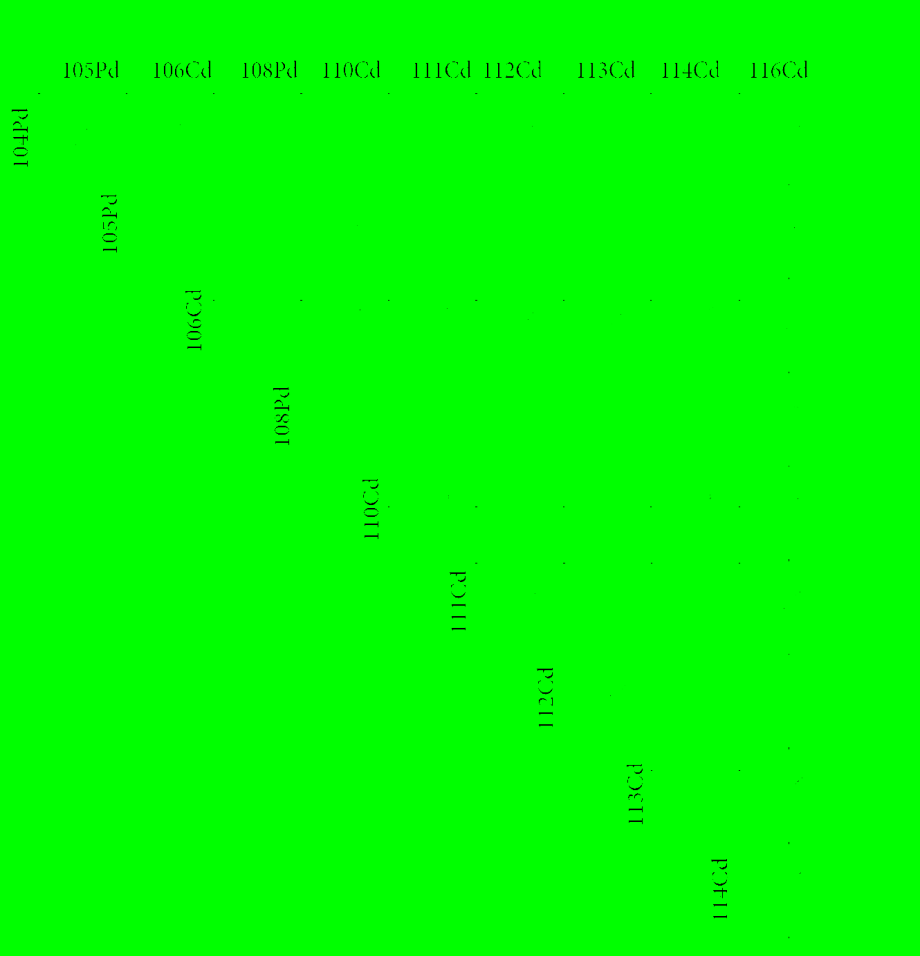
**

**Supplementary** **Figure 3. 10-choose-2 barcoding scheme. (A)** Staining indices for 5 Cd-tagged antibodies when they are used alone or in combinations of 2 and 3 antibodies. (**B**) Two-dimensional t-SNE maps in Figure 3D are colored for 10 Cd or Pd-tagged CD45 antibodies. (**C**) Pooled samples are deconvoluted using Premessa R package and de-barcoded samples are shown per assigned barcoding tags (n=45). (**D**) Purity matrix showing fractional abundance of samples in each deconvoluted sample.

**
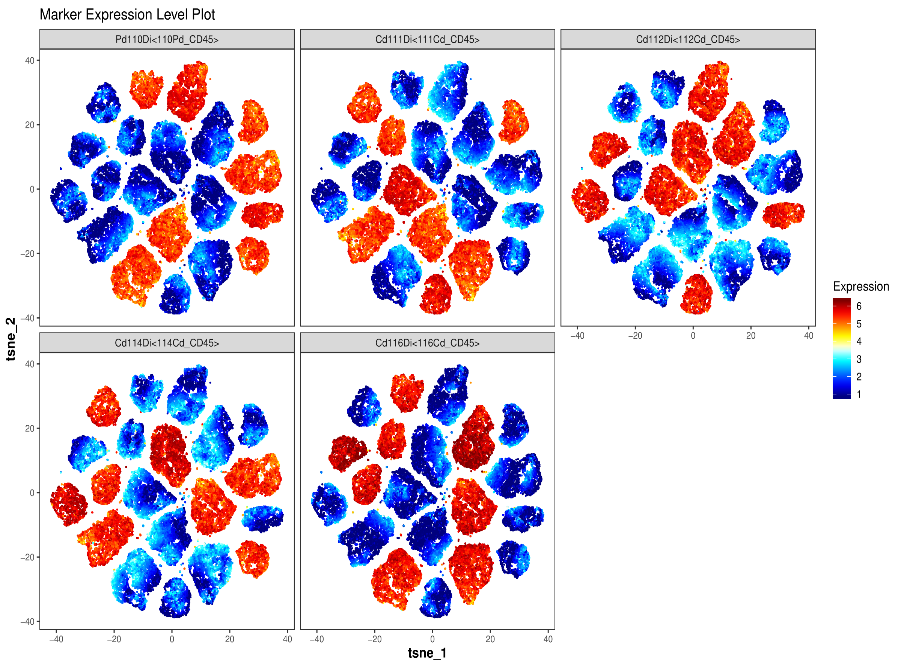

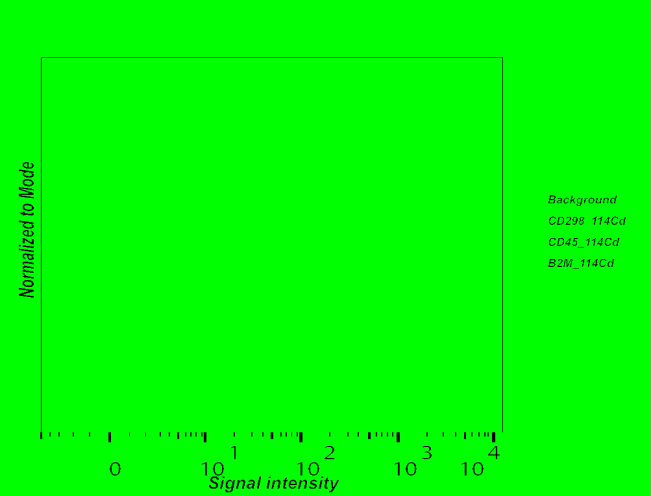
**

**B**

**A**

**Figure S4**

**C**

**E**

**D**

**
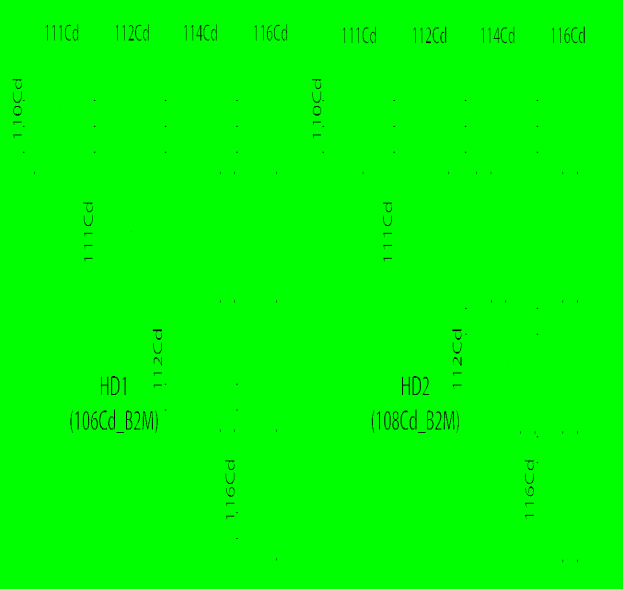

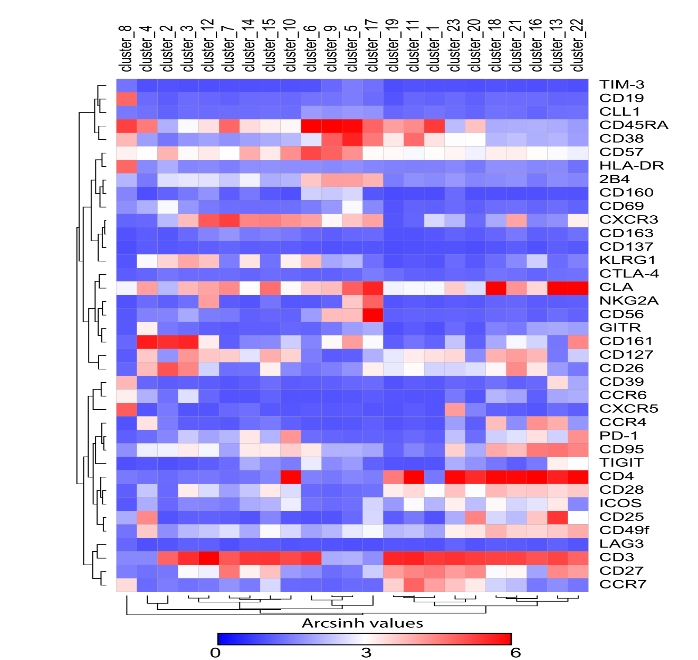

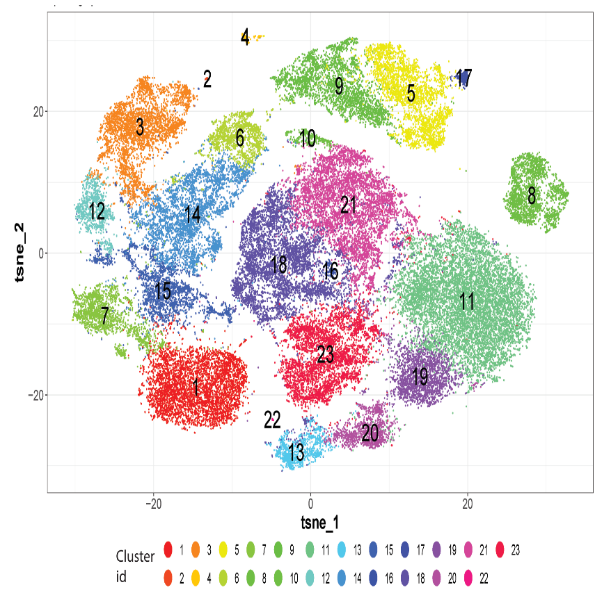
**

**Supplementary** **Figure 4. Utilizing live-cell barcoding to assess T-cell Phenotypes.** (**A**) Histograms show signal intensities of CD298 (purple), CD45 (green), and B2M (yellow) on PBMCs and CD298 (purple), CD45 (green), and B2M (yellow) antibodies tagged with 114Cd were used. Gray histogram background signal intensity. **(B)**t-SNE maps in Figure 4A are colored based on expression of 5 CD45-based tags used for sample barcoding. Scale shows arcsinh values. **(C)** Deconvoluted samples from two HDs (left, HD1 and right HD2) are plotted per assigned barcoding tags. (**D)** PhenoGraph clusters observed in deconvoluted samples from 2 HDs in Figure 4C (**E**) Heatmap shows marker expression levels for 23 PhenoGraph clusters in (D). Arcsinh values are shown.
